# Conditional Knockout of Raptor/mTORC1 Results in Dentin Malformation

**DOI:** 10.3389/fphys.2019.00250

**Published:** 2019-03-29

**Authors:** Furong Xie, Qinggang Dai, Xiao Liu, Jun Wang

**Affiliations:** ^1^ Department of Pediatric Dentistry, Shanghai Ninth People’s Hospital, College of Stomatology, Shanghai Jiao Tong University School of Medicine, Shanghai, China; ^2^ National Clinical Research Center for Oral Diseases, Shanghai, China; ^3^ Shanghai Key Laboratory of Stomatology, Shanghai Research Institute of Stomatology, Shanghai, China

**Keywords:** dentinogenesis, tooth abnormalities, odontoblast, S6 kinase 1, gene knockout techniques, RNA sequence analysis

## Abstract

mTORC1 signaling plays an important role in extracellular and intracellular signals, including growth factors, nutrients, energy metabolism, and stress. However, the functional role of mTORC1 in dentinogenesis is unknown. To study the role of Raptor/mTORC1 in dentinogenesis, an Raptor^fl/fl^; Osx-Cre (Rap-Osx) mouse, in which Raptor was conditionally deleted in odontoblasts and dental mesenchymal cells, was generated, and postnatal tooth development was compared between Rap-Osx mice and control littermates. Rap-Osx mice presented a phenotype known as dentinogenesis imperfecta and had smaller tooth volume, a thinner dentin layer and a larger pulp chamber. The proliferation and differentiation of odontoblasts/preodontoblasts were attenuated in mutant mice, which was likely responsible for the defects in dentinogenesis. Raptor/mTORC1-pS6K1 signaling was inactivated during tooth development in Rap-Osx mice, whereas it was activated in control mice. These results indicate that Raptor/mTORC1 plays a critical role in dentinogenesis *via* promoting odontoblasts/preodontoblasts proliferation and differentiation. Raptor/mTORC1 might regulate tooth development through the pS6K1 signaling pathway.

## Introduction

Proper development of dentin tissue is critical for tooth function, and deficits in dentinogenesis can cause various tooth disorders, such as dentinogenesis imperfecta type II and dentin dysplasia. In the early stage of development, odontoblasts differentiate from neural crest cells and play an important role in dentinogenesis during and after tooth eruption (Tatullo et al., 2015; Kawashima and Okiji, 2016). Odontoblasts secrete and mineralize hydroxyapatite crystals to retract and form a single-cell layer at the outermost region of the dental pulp. In the long cellular process of extension, odontoblasts extend deep into the dentin layer (Bleicher, 2014). During the period of odontoblast differentiation, multiple signaling pathways are integrated in the cell and influence the fate of odontoblasts (Sagomonyants and Mina, 2014; Yun et al., 2016; Ishikawa et al., 2017; Sagomonyants et al., 2017). However, the spatial and temporal regulation of tooth development is still unclear.

The mammalian/mechanistic target of rapamycin (mTOR) is an evolutionarily conserved protein kinase. There are two mTOR multiprotein complexes that differ in structure and function: mTORC1 and mTORC2 (Bhaskar and Hay, 2007; Zoncu et al., 2011; Del Fattore et al., 2012). Raptor is the key component of mTORC1 that distinguishes it from mTORC2 and thus results in unique downstream targets. One of the important mTORC1 downstream targets is S6 kinase 1 (S6K1), which phosphorylates a series of substrates, including S6 ribosomal protein (S6). Through phosphorylating these substrates, S6K1 can regulate gene transcription, protein synthesis, and other biological processes (Um et al., 2004; Yamnik et al., 2009; Carnevalli et al., 2010; Bahrami et al., 2014). 4E-BP1, which is a translation inhibitor, is also an important downstream target and can promote protein translation after phosphorylation by activated mTORC1 (Martelli et al., 2011). mTORC1 signaling is a critical component of several cellular signals, such as growth factors, nutrients, energy metabolism, and stress. It also has been a potential drug target for treating diseases, including renal cell carcinoma, mantle cell lymphoma, and tuberous sclerosis (Voss et al., 2011; Budde and Gaedeke, 2012; Smith, 2012). Recent studies have shown that mTORC1 might play a critical role in odontoblast differentiation. In human exfoliated deciduous teeth (SHED) cell experiments, Kim et al. (2011) reported that the inhibition of mTORC 1 *via* reduced expression of Raptor severely reduced the synthesis of dentin sialoprotein (DSP) and decreased the deposition of a mineralized matrix. Eapen and George (2015) observed that activation of mTOR signaling pathways led to activation of transcription factor nuclear factor κB (NF-κB) and thus promoted terminal differentiation of preodontoblasts during *in vitro* rat immortalized preodontoblast cell experiments. However, further *in vivo* studies are needed to confirm the results of the *in vitro* experiment and the specific mechanism involved.

Our previous study revealed that Raptor affected bone development by promoting osteoblast differentiation (Dai et al., 2017b). In the present study, we generated Raptor/mTORC1 conditional knockout mice to investigate the relationship between mTORC1 signaling and dentinogenesis.

## Materials and Methods

### Mice

mTOR^fl/fl^ mice bearing loxP sites flanking exons 1–5 of the mTOR gene (Stock No: 011009) and Rap^fl/fl^ mice bearing loxP sites flanking exon 6 of the Raptor gene (Stock No. 013188) were kind gifts from Prof. Tong. mTOR^fl/fl^ mice were crossed with Osx-Cre mice to generate mTOR^fl/fl^; Osx-Cre (mTOR^Osx/fl^) and mTOR^fl/+^; Osx-Cre (mTOR^Osx/+^) mice to identify the functional role of the mTOR complex in dentinogenesis. Then, Rap^fl/fl^ mice were crossed with Osx-Cre mice to generate Raptor^fl/fl^; Osx-Cre (Rap^Osx/fl^) and Raptor^fl/+^; Osx-Cre (Rap^Osx/+^) mice to clarify mTORC1, a component of the mTOR complex that plays an important role in dentinogenesis. The primers for genotyping are listed in Supplementary Table 1. All mice were bred and maintained under specific pathogen-free (SPF) conditions, and all experiments were performed according to a protocol approved by the Animal Care and Use Committee of the Shanghai Ninth People’s Hospital, Shanghai Jiao Tong University School of Medicine.

### Micro-CT Analysis

Mandibles with molars and incisors were harvested from 4-week-old mice for micro-CT scanning (SCAN 1176, Bruker, Kontich, Belgium). Before micro-CT scanning, all samples were kept in 70% ethanol. The middle coronal section of the mesio-root and the middle sagittal section of the mandibular first molars were used to analyze the dentin thickness. The whole tooth was used to analyze the dentin percentage. CT-volume software from Bruker was used to analyze the tooth volume, dentin percentage, and dentin width.

### Immunohistochemistry, Immunofluorescence, and Double Fluorochrome Labeling

The mandibles from Raptor conditional knockout and control mice were decalcified in 10% ethylenediaminetetraacetic acid (EDTA, pH 7.4) at 4°C for 1 month. The specimens were dehydrated through a graded ethanol series, embedded in paraffin, cut into 4-μm-thick sections, and then analyzed by hematoxylin and eosin (H&E) staining. Primary antibodies used in the experiments included mouse polyclonal anti-Ki67 antibody (gift from Prof. Tong), Raptor, mTOR (1:200, Lifespan BioSciences, Inc., Seattle, USA), p-S6K1, p-S6 (1:200, Merck Millipore, Darmstadt, Germany) Dspp, and OCN (Santa Cruz Biotechnology, Inc., California, USA). PBS solution was incubated as negative control (Supplementary Figure 1). For immunofluorescence staining, the sections were incubated with a fluorescence-labeled secondary antibody (1:500, Jackson) and stained with DAPI. For immunohistochemical staining, the sections were incubated with a horseradish peroxidase-labeled secondary antibody followed by color development with DAB (Gene Tech, Shanghai, China). The integrated optical density (IOD)/area showed the mean optical density of the dye staining. For double fluorescent labeling, calcein (5 mg/kg; Sigma-Aldrich) was first intraperitoneally injected into 2-week-old mice, followed by injection of an Alizarin red label (20 mg/kg; Sigma-Aldrich) 7 days later (Dai et al., 2017a).

### Backscattered and Resin-Casted Scanning Electron Microscopy (SEM)

The mandibles from all groups of 4-week-old mice for SEM were treated as previously reported (Gibson et al., 2013). The samples were dissected and fixed in 2% paraformaldehyde and 2.5% glutaraldehyde in a 0.1 M cacodylate buffer solution (pH 7.4). They were then dehydrated in ascending percentages of ethanol, embedded in Epon812 resin and oriented to expose the mandibular first molar region. Microcloths with Metadi Supreme polycrystalline diamond suspensions of decreasing sizes (Buehler, USA) were used to polish the surfaces of the samples. The samples were coated with gold for backscattered scanning electron microscopy analysis. For resin-casted scanning electron microscopy, the dentin surface was acid etched (37% phosphoric acid, 2–10 s), washed with 5.25% sodium hypochlorite (5 min), and coated with gold. Then, the samples were observed using an FEI Company Quanta 250 field emission environmental scanning electron microscope (Hillsboro, OR, USA).

## Cell Culture

The disassociated first molars were digested in dispase II (1 mg/ml) (Thermo Fisher, MA, USA) for 15 min at 37°C. Then, the enamel organs were removed while the dental papilla was digested in dispase II for another 15 min at 37°C. After centrifugation at 500 × g for 5 min, dental mesenchymal cells were suspended in α-MEM with 10% FBS and 1% penicillin/streptomycin and cultured in 24-well plates. After culturing for 10 days, the dental papilla cells were reseeded at 2.5 × 10^5^/cm^2^ and cultured in osteogenic medium (α-MEM with 10% FBS, 1% penicillin/streptomycin, 100 nM dexamethasone, 50 μM l-ascorbic acid, and 10 mm β-glycerophosphate) for 21 days. Mineralized nodule formation was detected by Alizarin red staining 21 days after osteogenesis differentiation (Cyagen Biosciences, CA, USA).

### RNA-seq and Quantitative PCR (qPCR)

Total RNA was extracted from odontoblasts differentiated from dental mesenchymal cells after 7 days using TRIzol (TaKaRa, Shiga, Japan) following a standard protocol (Invitrogen, Carlsbad, CA, USA). An aliquot of 500 ng of RNA was reverse transcribed to cDNA using TaKaRa PrimeScript Reverse Transcriptase (TaKaRa, Shiga, Japan). qPCR was performed using a SYBR green mixture (TaKaRa, Shiga, Japan). The primers used for qPCR are listed in Supplementary Table 2. The relative expression levels of the genes of interest were analyzed by 2^−ΔΔC^. Complementary DNAs (cDNAs) were synthesized from TRIzol (Invitrogen, Carlsbad, CA, USA)-isolated RNA using Superscript III kits (Invitrogen, Carlsbad, CA, USA). RNA-seq libraries were prepared and sequenced at Shanghai OE Biotech Inc. (Shanghai, China). Sequencing reads were mapped by TopHat and transcripts called by Cufflinks.

### Western Blot

Dental papilla tissues from control, Rap-Osx mice and dental mesenchymal cells cultured with p-S6K1 inhibitor (PF-4708671, Selleck chemicals, S2163) were collected for western blot. For the cell culture, DMSO was used as a control solution. Total proteins were extracted using 1× SDS lysis buffer (TaKaRa Bio Inc., Shiga, Japan) containing a protease inhibitor cocktail. The lysates were centrifuged at 14,000 g for 10 min at 4°C, and the supernatants containing the proteins were collected. Lysates containing equal protein (20 μg/lane) were separated by 10% SDS-PAGE followed by western blotting according to a standard protocol. β-Actin antibody was purchased from Sigma, and the other antibodies used for p-S6K1, p-S6, and DSPP are listed in the Immunohistology section.

### Statistical Analysis

All quantitative data are presented as the mean ± SD from at least three independent samples. A value of *p* < 0.05 (two-tailed Student’s *t*-test) was considered statistically significant. GraphPad Prism 5.0 software was used for data analysis.

## Results

### Raptor/mTORC1 Conditional Knockout Resulted in Defects in Tooth Development

**Figure 1:**
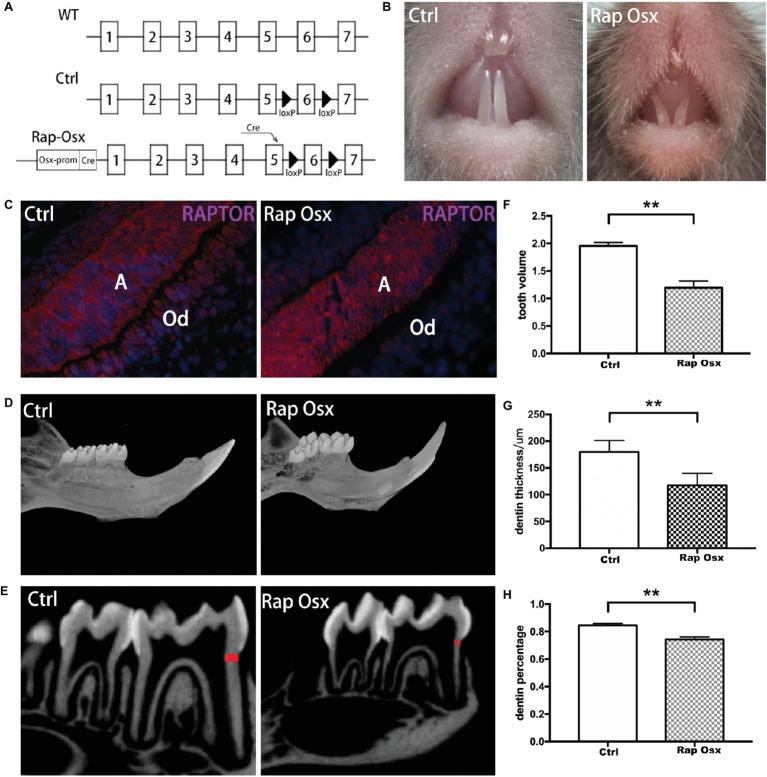
Conditional knockout of Raptor resulted in defects in dentinogenesis. **(A)** Diagram of the breeding strategy used to obtain Osx-Cre mice with mediated homozygous deletion of Raptor (Rap-Osx) (WT: wild-type mouse; Ctrl: WT mouse with exon 6 flanked by two LoxP sites; white tile: exon; black arrowhead: LoxP site). **(B,C)** Immunostaining of the Raptor protein in odontoblasts/preodontoblasts of the Ctrl and Rap-Osx mice, respectively. **(D)** 3D reconstruction of the mandible and molars of the Ctrl and knockout mice. **(E)** Sagittal section of micro-CT scanning of the lower first molars of Ctrl and Rap-Osx mice; the dentin, labeled in red, was used for dentin thickness analysis. **(F–H)** Statistical analysis of the tooth volume, dentin thickness and dentin percentage of the Ctrl and knockout mice. Data represent the mean ± SD (**p < 0.001). A, ameloblasts; Od, odontoblasts.

We first generated mTOR conditional knockout mice to observe the functional role of mTOR in dentinogenesis. The results showed that dentin thickness decreased, and the pulp chamber enlarged significantly in mTOR^fl/fl^; Osx-Cre mice (Supplementary Figure 2). Then, a mouse in which Raptor was conditionally deleted in odontoblasts and dental mesenchymal cells was generated to explore the role of mTORC1 signaling in dentinogenesis. A diagram of the breeding strategy is shown in Figure 1A. The immunofluorescence staining results showed that Raptor was almost completely knocked out in the odontoblast cell layer but was still expressed in the ameloblast cell layer (Figure 1C). This result indicated that Raptor expression in odontoblasts was dramatically reduced, whereas its expression in ameloblasts was not influenced. Inactivation of mTORC1 in odontoblasts resulted in impaired tooth development (Figure 1B). Micro-CT scanning 3D reconstruction results showed that the mandibular incisors and molars of Rap-Osx mice were much smaller than those of control mice at P28 (Figure 1D); a shorter root, larger pulp cavity, and less dentinogenesis were also observed (Figure 1E). These results indicate that mTORC1 plays an important role in dentinogenesis.

### Abnormal Formation of Dentin in Raptor Conditional Knockout Mice

**Figure 2:**
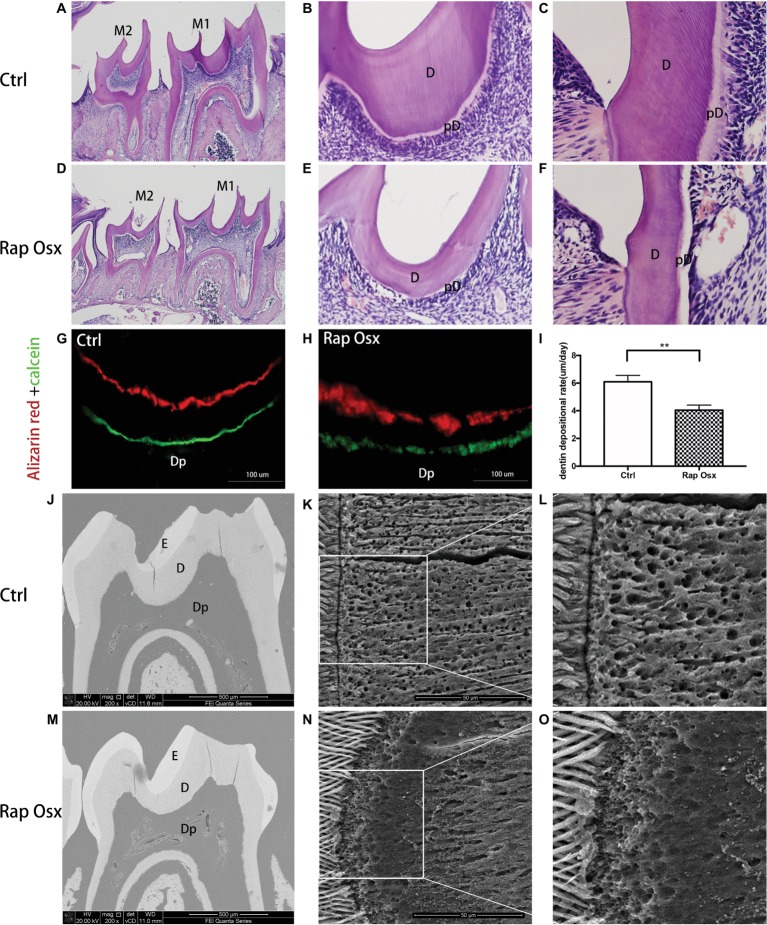
Analyses of the tooth tissue, dentin mineralization level and dentinal tubular structures of Rap-Osx mice. **(A–F)** HE staining of the mandibular first molars from Ctrl mice and Rap-Osx mice. Mature dentin and predentin were both thinner in Rap-Osx mice than in the control group, while the pulp chambers were enlarged in Rap-Osx mice. **(G–I)** Alizarin and calcein double fluorochrome labeling was performed to determine the dentin apposition rate of 4-week-old control and mutant mice. Mice were first injected with calcein (green). Two weeks later, they were injected with Alizarin red (red). Representative images were captured of the mandibular first molar dentin region at the position of the middle sagittal and coronal section. The distance between these two labels represented the dentin matrix deposited over a period of 14 days. The labels for the mutant mice were thinner (Scale bars = 100 μm). Statistical analyses of the dentinogenesis rate were performed. **(J–O)** Backscattered scanning electron microscopy images show the enamel in white, dentin in gray, and low mineral-density areas in black. The mutant molars had irregular dentin with several black/dark areas (M), indicating the hypomineralization of mutant molars. **(K,L)** Resin-casted and acid-etched scanning electron microscopy analyses of the dentinal tubules of Ctrl mice. **(N,O)** Resin-casted and acid-etched scanning electron microscopy analyses of the dentinal tubules of mutant mice. E, enamel; D, dentin; pD, predentin; Dp, dental pulp. Note that the dentinal tubules of the Raptor cKO mice were unevenly distributed. Scale bars = 50 μm. Data represent mean ± SD (**p < 0.001).

First mandibular molar samples were harvested from mice at 7, 14, 21, and 28 days after birth and analyzed. The dentin of the first mandibular molars was well formed in the control mice with uncalcified predentin deposited adjacent to dental papilla cells. However, inactivation of Raptor in the mesenchymal cells of the dental papilla resulted in defective dentinogenesis. Smaller tooth volume was observed in P28 Rap-Osx mice. Moreover, the dentin width and percentage were significantly decreased in P28 Rap-Osx mice compared with control mice (Figures 1F–H). As shown in Figures 2A–F, H&E staining also revealed a decreased dentin and predentin thickness in Rap-Osx mice. Next, we analyzed the predentin/dentin mineralization and dentinal tubular structures in the two groups of P28 mice. Double fluorochrome labeling was employed to assess the dentin deposition rate. The amount of newly generated dentin between two fluorescent labels was reduced in the Rap-Osx mice (*n* = 3 per group) (Figures 2G–I). In addition, no significant difference in dentin mineralization was observed according to the backscattered scanning electron microscopy analysis (Figures 2J,M). However, a resin-casted scanning electron microscopy analysis was used to examine the dentinal tubular structures. In mutant mice, the tubules appeared completely disorganized (Figures 2N,O), in contrast to the well organized and evenly distributed tubules in the control mice (Figures 2K,L). Together, these results demonstrate that knockout of Raptor expression resulted in predentin/dentin mineralization defects and abnormal dentinal tubules.

### Raptor Promoted Odontoblast/Preodontoblast Proliferation and Differentiation

**Figure 3:**
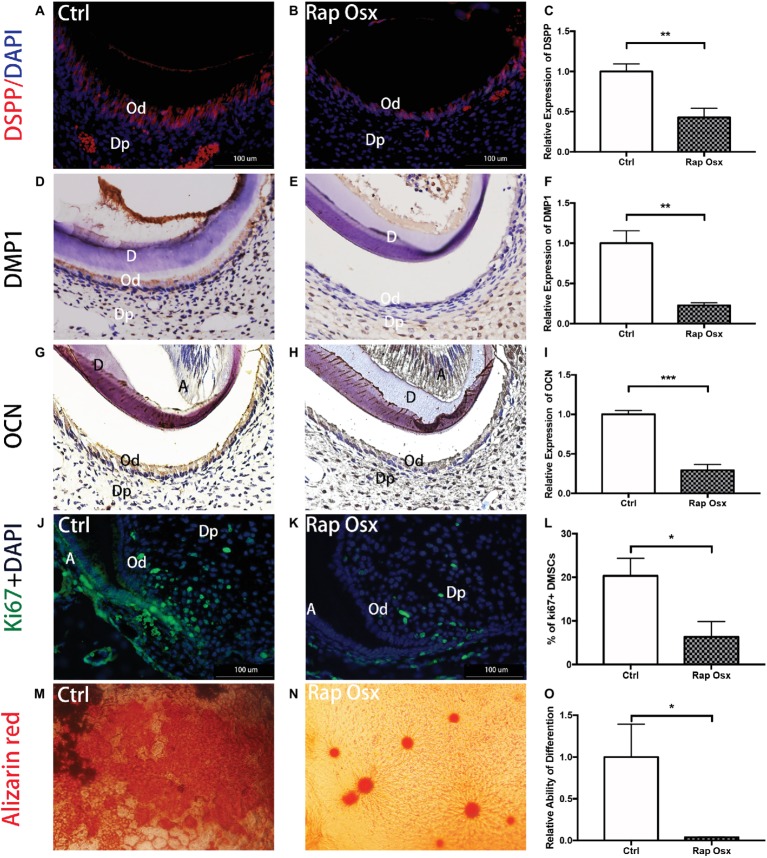
Rap-Osx mice showed repressed odontoblast proliferation, differentiation and DSPP secretion. **(A,B)** The protein expression of DSPP in developmental teeth of Rap-Osx mice and Ctrl mice. **(D,E)** The protein expression of DMP1 in developmental teeth of Rap-Osx mice and Ctrl mice. **(G,H)** The protein expression of OCN in developmental teeth of Rap-Osx mice and Ctrl mice. **(J,K)** The protein expression of Ki67 in developmental teeth of Rap-Osx mice and Ctrl mice. **(C,F,I)** The protein expression levels of DSPP, DMP1 and OCN in developmental teeth of Rap-Osx mice and Ctrl mice. **(L)** Quantitative analysis of the Ki67 positive dental mesenchymal cells in developmental teeth of Rap-Osx mice and Ctrl mice. **(M,N)** Alizarin red staining was performed on primary cultured dental mesenchymal cells from Ctrl and Rap-Osx mice after osteogenesis induction for 21 days. **(O)** Quantitative analysis of the relative differentiation ability, i.e., calcium knot formation ability. Data represent the mean ± SD (*p < 0.05, **p < 0.001, ***p < 0.0001).

We detected the protein expression level of dentin sialophosphoprotein (DSPP), DMP1, and OCN in the tooth; the results showed that DSPP, DMP1, and OCN levels in Rap-Osx mice were decreased in the dentin and predentin tissue and odontoblasts, whereas the levels in control mice were not affected (Figures 3A–I). This result suggests a functional requirement of mTORC1 signaling in odontogenic differentiation in dental mesenchymal cells. The dentin phenotype of Rap-Osx mice prompted us to investigate why the secretion of dentin matrix by odontoblasts was decreased in conditional knockout mice. We also aimed to determine whether the Raptor protein could promote the proliferation and differentiation of dental mesenchymal cells. First, we detected the expression level of Ki67, which is indicative of the proliferation of dental mesenchymal cells. The results revealed that the Ki67-positive dental mesenchymal cells were less detectable in conditional knockout mice (Figures 3J–L). Next, we cultured primary dental papilla cells from Rap-Osx and control mice at the age of P7 and analyzed the proliferation and differentiation abilities of dental papilla cells. Our data indicated that the dental mesenchymal cells from Rap-Osx mice exhibited decreased calcification (Figures 3M–O) and proliferation abilities (Supplementary Figure 3). These *in vitro* experiments support a positive role of Raptor in odontogenesis through the promotion of odontoblast differentiation and dental mesenchymal cells proliferation, hence affecting the rate of dentin matrix protein synthesis.

### Differentiation and Cell Proliferation-Related Genes Were Downregulated in Mutant Odontoblasts

**Figure 4:**
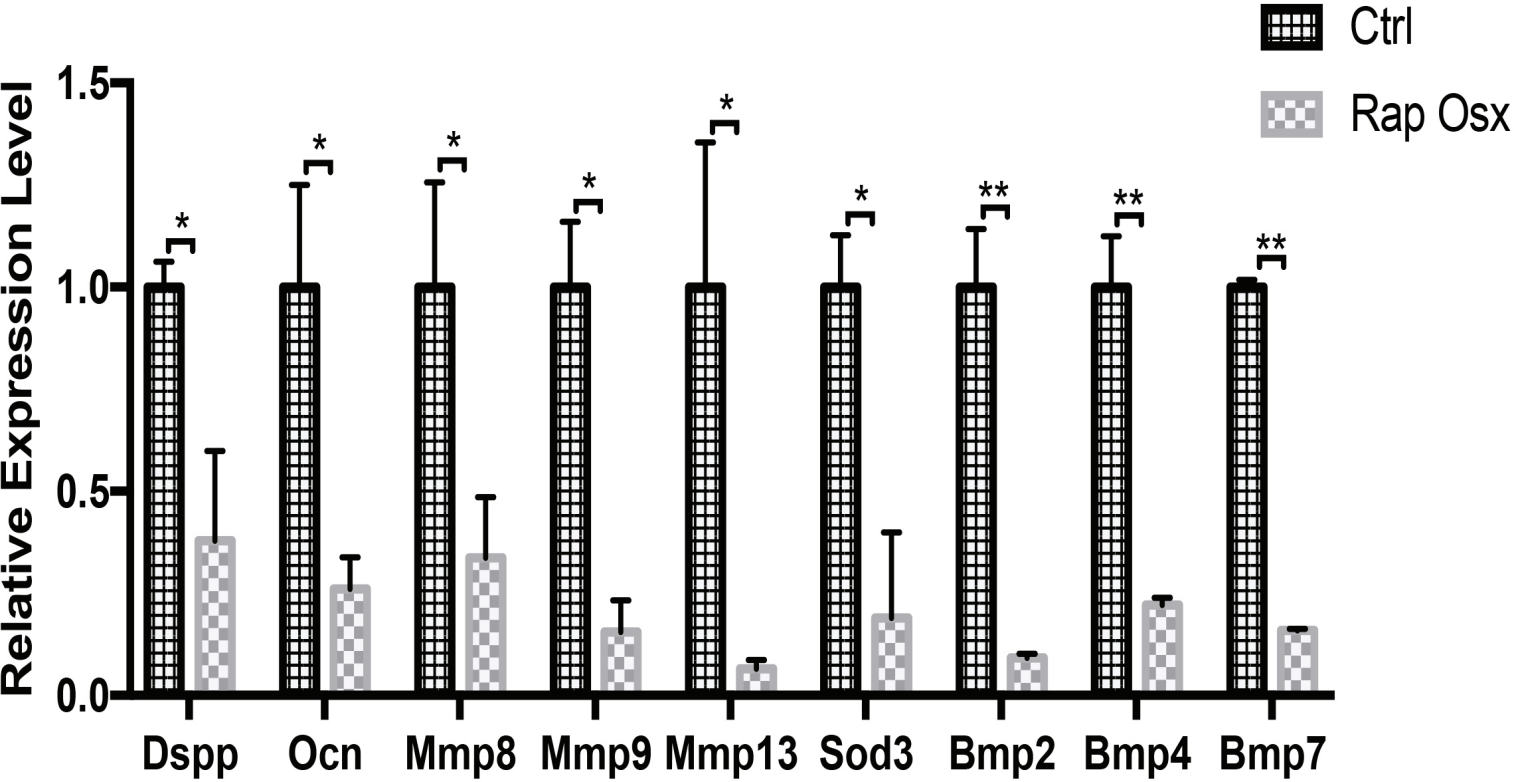
Expression levels of selected genes were validated by qPCR (*p < 0.05, **p < 0.001).

To further identify the downstream targets of mTORC1 signaling involved in dentinogenesis, we analyzed gene expression profiles in the dental papilla tissue of the molar mesenchyme from P7 Rap-Osx and control mice using RNA-seq. We identified 182 genes that were differentially expressed in Rap-Osx versus control mice (FDR *p* < 0.05, *n* = 3). The heat maps of RNA-seq are shown in Supplementary Figure 4A. Of these 182 genes, 119 were downregulated, and 63 were upregulated, as indicated by representative mouse genome screen shots. A pathway enrichment assay demonstrated that, in addition to the cAMP pathway in which mTORC1 is involved with target genes (Chang et al., 2015), genes related to carbon metabolism in cancer, nicotine addiction, morphine addiction, renin secretion, insulin secretion, dopaminergic synapses, GABAergic synapses, cholinergic synapses, retrograde endocannabinoid signaling, cell adhesion molecules, neuroactive ligand-receptor interaction, and the calcium signaling pathway were among the most enriched categories (Supplementary Figure 4B). Moreover, certain genes related to the extracellular matrix and mineral tissue development were selected for further analysis by qPCR. Consistent with the RNA-seq results, we found that these genes were dysregulated, which demonstrated that the results of the RNA-seq were reliable (Figure 4). However, no significant difference was observed in Runt-related transcription factor 2 (Runx2), which was reported dysregulated in our previous study (Dai et al., 2017b; Supplementary Figure 5).

### Raptor Promoted Dentinogenesis Through Stimulation of S6K1 Phosphorylation

**Figure 5:**
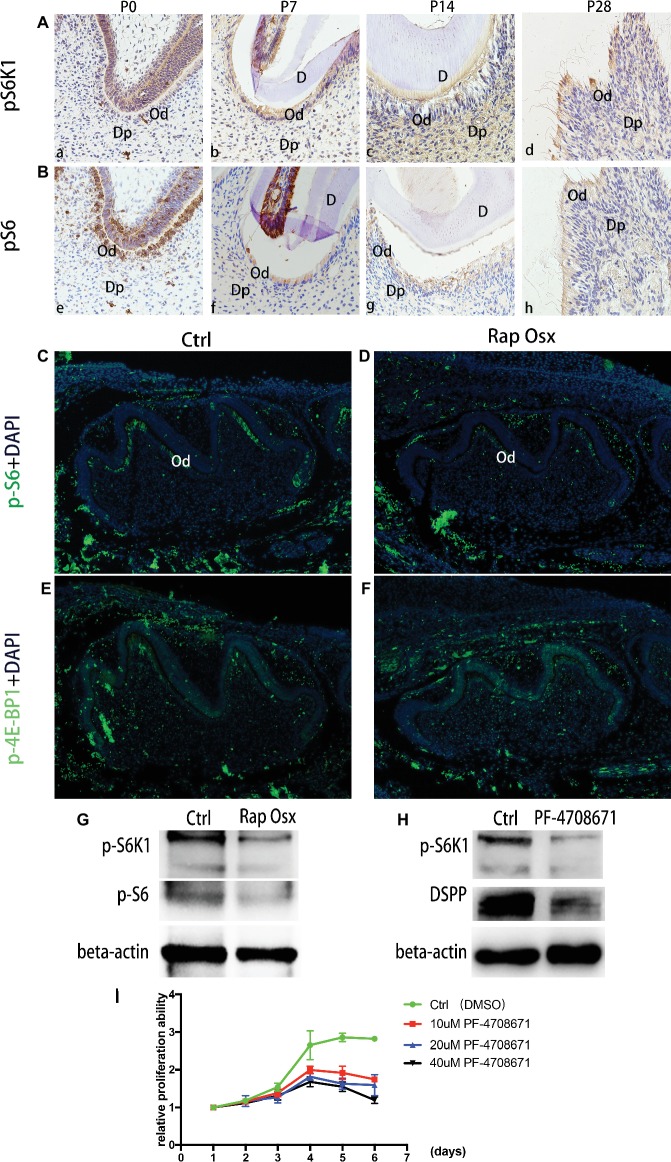
Phosphorylation capacity of pS6K1 decreased after Raptor deficiency. **(A)** Detection of phosphorylation levels of pS6K1 in the mandibular first molar was performed by immunohistochemistry staining at P0 (a), P7 (b), P14 (c), and P28 (d). **(B)** Detection of the phosphorylation levels of pS6 in the mandibular first molar was performed at P0 (e), P7 (f), P14 (g), and P28 (h). **(C,D)** Detection of phosphorylated S6 in the mandibular first molar at P0. **(E,F)** Detection of phosphorylated 4E-BP1 in the mandibular first molar at P0. DP, dental pulp; OB, odontoblasts; D, dentin. **(G)** Protein expression levels of p-S6K1 and p-S6 in WT and mutant mice. **(H)** The DSPP expression level decreased by the S6K1 inhibitor. **(I)** PF-4708671 inhibited the proliferation of dental mesenchymal cells from WT mice.

S6K1 is an important downstream effector molecule of the mTORC1 pathway (Bahrami et al., 2014). Our recent study showed that activation of the mTORC1-S6K1 signaling pathway plays a vital role in osteoblast and osteoblast differentiation (Dai et al., 2017b), but it remains unclear whether the mTORC1-S6K1 axis functions in odontoblast differentiation. First, we analyzed activation of the mTORC1-S6K1 pathway during dentinogenesis by detecting phosphorylated S6K1 expression as a readout. At E18.5 and prior to the initiation of dentinogenesis, S6K1 phosphorylation was almost undetectable. At P0, S6K1 showed a high phosphorylation level in the mandibular first molar (Figures 5A,B). From P7 to P28, the phosphorylation level of S6K1 was gradually downregulated and restricted to the apical region. A similar expression pattern of S6, one of the key substrates of S6K1, was observed from the late bell development period to adulthood by immunohistochemistry staining. Collectively, these results indicate that the mTORC1-S6K1 pathway is involved in dentinogenesis. Therefore, we hypothesized that S6K1 phosphorylation ability may be altered due to Raptor deficiency, which eventually results in malformation of the tooth. Then, immunofluorescence staining experiments were conducted, which showed that phosphorylated S6K1 protein was downregulated in Rap-Osx mice, while phosphorylated 4E-BP1 was not significantly different (Figures 5C–F). Moreover, the results of western blot experiments proved that phosphorylated S6K1 was decreased in the mutant mice (Figure 5G). The dental mesenchymal cells from WT mice were also cultured *in vitro*, and S6K1 inhibitor (PF-4708671) was added to the culture medium. The results showed that the expression level of DSPP was downregulated, and the proliferation abilities of primary cultured dental mesenchymal cells decreased with increasing concentration of S6K1 inhibitor (Figures 5H,I). Together, the results indicate that S6K1 phosphorylation ability can be altered due to Raptor deficiency, which results in the downregulation of DSPP, and eventually affects dentinogenesis.

## Discussion

In this study, we revealed the important role of mTOR/Raptor signaling in odontoblast differentiation and dentinogenesis by inducing conditional knockout of mTOR and Raptor, respectively. First, deletion of mTOR in preodontoblasts resulted in obvious dentin defects with thinner dentin formation. The manifestation was in accordance with the conclusion that mTOR is essential for dentin formation. Furthermore, deletion of Raptor, the unique element of mTORC1, from preodontoblasts reproduced almost all dentin phenotypes of mTOR-Osx mice. This finding indicated that mTORC1 is the main functional complex of mTOR in odontoblasts. Our results showed that blockage of mTORC1 signaling *via* deletion of either mTOR or Raptor resulted in dentin defects, indicating that Raptor/mTORC1 signaling may promote dentinogenesis. Kim et al. (2011) described a decrease in calcification in Raptor-inhibited dental pulp stem cells. Their study strongly suggested an important role of mTORC1 in dental mesenchymal cell differentiation and mineralization. Based on conditional knockout of Raptor *in vivo*, our study directly supports the hypothesis of the previous study. Moreover, on the basis of this finding, our study focused on the precise mechanism of Raptor/mTORC1 signaling in regulating odontoblast differentiation and dentinogenesis.

In our study, several lines of evidence indicated that the inactivation of mTORC1 can influence odontoblast differentiation and dentinogenesis. First, odontoblast-specific knockout of Raptor in mice obviously reduced the expression of odontoblast markers, such as DSPP and OCN. Second, the dentinogenesis rate, which was measured by Alizarin red/calcein labeling, was reduced in Rap-Osx mice compared with control mice. Third, an *in vitro* study showed that odontoblast differentiation of dental mesenchymal cells from Rap-Osx mice was impaired. Collectively, our findings demonstrate that Raptor/mTORC1 signaling is essential for odontoblast differentiation and dentinogenesis.

The Dspp gene encodes three principal proteins of the dentin extracellular matrix of the tooth. Odontoblasts secrete preprotein, which is eventually cleaved into dentin sialoprotein (DSP), dentin phosphoprotein (DPP), and dentin glycoprotein (DGP) (Yamakoshi et al., 2005; von Marschall and Fisher, 2010). These proteins become the major non-collagenous proteins of dentin and play an important role in the regulation of biomineralization process of dentin (Yamakoshi, 2008; Koike et al., 2014; Villarreal-Ramirez et al., 2017). Mutations of Dspp have been reported to associate with dentinogenesis imperfecta-II (Zhang et al., 2001; Sreenath et al., 2003; Kim et al., 2005), and allelic differences due to repeat polymorphisms have been found for this gene. However, many patients with dentinogenesis imperfecta have no Dspp gene mutation, which may imply that other mechanisms are involved in dentinogenesis imperfecta. Our results showed that the DSPP protein was downregulated in the mandibular first molar of Raptor knockout mice compared with control mice. A similar expression pattern was also observed for DMP1 protein, which is also critical for dentinogenesis (Rangiani et al., 2012). These phenomena were similar to the results reported by Kim et al. (2011).

The precise mechanism by which Raptor/mTORC1 signaling promotes odontoblast differentiation and dentinogenesis is still unknown. We found that the differences in DSPP and DMP1 protein expression levels were more obvious than the differences in gene expression levels, which indicated that mTORC1 might enhance dentinogenesis by promoting protein synthesis. S6K1, which is a direct downstream factor of mTORC1, may positively regulate the differentiation of both chondrocytes and adipocytes (Feng and Qiu, 2018; Xiong et al., 2018). The current study provided evidence that the mTOR/Raptor-S6K1 axis might participate in odontoblast differentiation. Interestingly, phosphorylation is more restricted in the preodontoblast/odontoblast layer than in other dental papilla tissue cells. This expression pattern may occur because dentinogenesis begins at birth as the space between the ameloblasts and odontoblasts widens. Immunohistochemistry and western blot experiments revealed a decreased level of phosphorylated S6 in odontoblast-specific Raptor knockout mice *in vivo*. Moreover, our previous study showed that S6K1 activity was reduced in Raptor-deficient bone marrow stem cells and was accompanied by impaired osteoblast differentiation (Dai et al., 2017b). Finally, no significant difference was found in the phosphorylation level of 4E-BP1 between control and mutant mice. S6K1 inhibitor suppressed the expression of DSPP. These results support our hypothesis that mTORC1 may promote dentinogenesis by enhancing the phosphorylation of S6K1 and S6.

In summary, our study identified the critical role of Raptor/mTORC1 signaling in odontoblast differentiation and dentinogenesis. Moreover, we revealed that the mTOR/Raptor-S6K1 axis might participate in the regulatory mechanism by regulating DSPP expression. This study provides new insights into dental dysplasias, such as dentinogenesis dysplasia, and provides a foundation for research on tooth regeneration and dental mesenchymal cells.

## Author Contributions

FX and JW contributed to the study conception, design, data acquisition, analysis, and interpretation and drafted and critically revised the manuscript. QD and XL contributed to the study conception, design, data acquisition, analysis, and interpretation and drafted the manuscript. All authors gave final approval and agreed to be accountable for all aspects of the work.

### Conflict of Interest Statement

The authors declare that the research was conducted in the absence of any commercial or financial relationships that could be construed as a potential conflict of interest.

## References

[ref1] Bahrami B. F. Ataie-Kachoie P. Pourgholami M. H. Morris D. L. (2014). p70 ribosomal protein S6 kinase (Rps6kb1): an update. J. Clin. Pathol. 67, 1019–1025. 10.1136/jclinpath-2014-202560, PMID: 25100792

[ref2] Bhaskar P. T. Hay N. (2007). The two TORCs and Akt. Dev. Cell 12, 487–502. 10.1016/j.devcel.2007.03.020, PMID: 17419990

[ref3] Bleicher F. (2014). Odontoblast physiology. Exp. Cell. Res. 325, 65–71. 10.1016/j.yexcr.2013.12.012, PMID: 24361392

[ref4] Budde K. Gaedeke J. (2012). Tuberous sclerosis complex-associated angiomyolipomas: focus on mTOR inhibition. Am. J. Kidney Dis. 59, 276–283. 10.1053/j.ajkd.2011.10.013, PMID: 22130643

[ref5] Carnevalli L. S. Masuda K. Frigerio F. Le Bacquer O. Um S. H. Gandin V.. (2010). S6K1 plays a critical role in early adipocyte differentiation. Dev. Cell 18, 763–774. 10.1016/j.devcel.2010.02.018, PMID: 20493810 PMC2918254

[ref6] Chang H. H. Young S. H. Sinnett-Smith J. Chou C. E. Moro A. Hertzer K. M.. (2015). Prostaglandin E2 activates the mTORC1 pathway through an EP4/cAMP/PKA- and EP1/Ca^2+^-mediated mechanism in the human pancreatic carcinoma cell line PANC-1. Am. J. Physiol. Cell Physiol. 309, C639–C649. 10.1152/ajpcell.00417.2014, PMID: 26310818 PMC4652080

[ref7] Dai Q. Xie F. Han Y. Ma X. Zhou S. Jiang L. (2017a). Inactivation of regulatory-associated protein of mTOR (Raptor)/mammalian target of rapamycin complex 1 (mTORC1) signaling in osteoclasts increases bone mass by inhibiting osteoclast differentiation in mice. J. Biolumin. Chemilumin. 292, 196–204. 10.1074/jbc.M116.764761 PMC5217679 27879318

[ref8] Dai Q. Xu Z. Ma X. Niu N. Zhou S. Xie F. (2017b). mTOR/Raptor signaling is critical for skeletogenesis in mice through the regulation of Runx2 expression. Cell Death Differ. 24, 1886–1899. 10.1038/cdd.2017.110 28686577 PMC5635215

[ref9] Del Fattore A. Teti A. Rucci N. (2012). Bone cells and the mechanisms of bone remodelling. Front. Biosci. (Elite Ed.) 4, 2302–2321. 10.2741/543 22202038

[ref10] Eapen A. George A. (2015). Dentin phosphophoryn in the matrix activates AKT and mTOR signaling pathway to promote preodontoblast survival and differentiation. Front. Physiol. 6:221. 10.3389/fphys.2015.00221, PMID: 26300786 PMC4528161

[ref11] Feng F. B. Qiu H. Y. (2018). Effects of Artesunate on chondrocyte proliferation, apoptosis and autophagy through the PI3K/AKT/mTOR signaling pathway in rat models with rheumatoid arthritis. Biomed. Pharmacother. 102, 1209–1220. 10.1016/j.biopha.2018.03.142, PMID: 29710540

[ref12] Gibson M. P. Zhu Q. Wang S. Liu Q. Liu Y. Wang X.. (2013). The rescue of dentin matrix protein 1 (DMP1)-deficient tooth defects by the transgenic expression of dentin sialophosphoprotein (DSPP) indicates that DSPP is a downstream effector molecule of DMP1 in dentinogenesis. J. Biolumin. Chemilumin. 288, 7204–7214. 10.1074/jbc.M112.445775, PMID: 23349460 PMC3591629

[ref13] Ishikawa Y. Nakatomi M. Ida-Yonemochi H. Ohshima H. (2017). Quiescent adult stem cells in murine teeth are regulated by Shh signaling. Cell Tissue Res. 369, 497–512. 10.1007/s00441-017-2632-x, PMID: 28547659

[ref14] Kawashima N. Okiji T. (2016). Odontoblasts: specialized hard-tissue-forming cells in the dentin-pulp complex. Congenit. Anom. 56, 144–153. 10.1111/cga.12169, PMID: 27131345

[ref15] Kim J. K. Baker J. Nor J. E. Hill E. E. (2011). mTor plays an important role in odontoblast differentiation. J. Endod. 37, 1081–1085. 10.1016/j.joen.2011.03.034, PMID: 21763898

[ref16] Kim J. W. Hu J. C. Lee J. I. Moon S. K. Kim Y. J. Jang K. T.. (2005). Mutational hot spot in the DSPP gene causing dentinogenesis imperfecta type II. Hum. Genet. 116, 186–191. 10.1007/s00439-004-1223-6, PMID: 15592686

[ref17] Koike T. Polan M. A. Izumikawa M. Saito T. (2014). Induction of reparative dentin formation on exposed dental pulp by dentin phosphophoryn/collagen composite. Biomed. Res. Int. 2014:745139. 10.1155/2014/745139, PMID: 24804241 PMC3997146

[ref18] Martelli A. M. Evangelisti C. Chappell W. Abrams S. L. Basecke J. Stivala F.. (2011). Targeting the translational apparatus to improve leukemia therapy: roles of the PI3K/PTEN/Akt/mTOR pathway. Leukemia 25, 1064–1079. 10.1038/leu.2011.46, PMID: 21436840

[ref19] Rangiani A. Cao Z. G. Liu Y. Voisey Rodgers A. Jiang Y. Qin C. L.. (2012). Dentin matrix protein 1 and phosphate homeostasis are critical for postnatal pulp, dentin and enamel formation. Int. J. Oral Sci. 4, 189–195. 10.1038/ijos.2012.69, PMID: 23258378 PMC3633060

[ref20] Sagomonyants K. Kalajzic I. Maye P. Mina M. (2017). FGF signaling prevents the terminal differentiation of odontoblasts. J. Dent. Res. 96, 663–670. 10.1177/0022034517691732, PMID: 28170285 PMC5444616

[ref21] Sagomonyants K. Mina M. (2014). Biphasic effects of FGF2 on odontoblast differentiation involve changes in the BMP and Wnt signaling pathways. Connect. Tissue Res. 55(Suppl. 1), 53–56. 10.3109/03008207.2014.923867 25158181 PMC4404504

[ref22] Smith S. M. (2012). Targeting mTOR in mantle cell lymphoma: current and future directions. Best Pract. Res. Clin. Haematol. 25, 175–183. 10.1016/j.beha.2012.04.008, PMID: 22687453

[ref23] Sreenath T. Thyagarajan T. Hall B. Longenecker G. D’Souza R. Hong S.. (2003). Dentin sialophosphoprotein knockout mouse teeth display widened predentin zone and develop defective dentin mineralization similar to human dentinogenesis imperfecta type III. J. Biolumin. Chemilumin. 278, 24874–24880. 10.1074/jbc.M303908200, PMID: 12721295

[ref24] Tatullo M. Marrelli M. Shakesheff K. M. White L. J. (2015). Dental pulp stem cells: function, isolation and applications in regenerative medicine. J. Tissue Eng. Regener. Med. 9, 1205–1216. 10.1002/term.1899, PMID: 24850632

[ref25] Um S. H. Frigerio F. Watanabe M. Picard F. Joaquin M. Sticker M.. (2004). Absence of S6K1 protects against age- and diet-induced obesity while enhancing insulin sensitivity. Nature 431, 200–205. 10.1038/nature02866, PMID: 15306821

[ref26] Villarreal-Ramirez E. Eliezer D. Garduno-Juarez R. Gericke A. Perez-Aguilar J. M. Boskey A. (2017). Phosphorylation regulates the secondary structure and function of dentin phosphoprotein peptides. Bone 95, 65–75. 10.1016/j.bone.2016.10.028, PMID: 27810285 PMC5234040

[ref27] von Marschall Z. Fisher L. W. (2010). Dentin sialophosphoprotein (DSPP) is cleaved into its two natural dentin matrix products by three isoforms of bone morphogenetic protein-1 (BMP1). Matrix Biol. 29, 295–303. 10.1016/j.matbio.2010.01.002, PMID: 20079836 PMC2862847

[ref28] Voss M. H. Molina A. M. Motzer R. J. (2011). mTOR inhibitors in advanced renal cell carcinoma. Hematol. Oncol. Clin. North Am. 25, 835–852. 10.1016/j.hoc.2011.04.008, PMID: 21763970 PMC3587783

[ref29] Xiong Y. Xu Z. Wang Y. Kuang S. Shan T. (2018). Adipocyte-specific DKO of Lkb1 and mTOR protects mice against HFD-induced obesity, but results in insulin resistance. J. Lipid Res. 59, 974–981. 10.1194/jlr.M081463, PMID: 29636366 PMC5983395

[ref30] Yamakoshi Y. (2008). Dentin sialophophoprotein (DSPP) and dentin. J. Oral Biosci. 50, 33–44. 10.2330/joralbiosci.50.33, PMID: 20037676 PMC2797732

[ref31] Yamakoshi Y. Hu J. C. Fukae M. Zhang H. Simmer J. P. (2005). Dentin glycoprotein: the protein in the middle of the dentin sialophosphoprotein chimera. J. Biolumin. Chemilumin. 280, 17472–17479. 10.1074/jbc.M413220200, PMID: 15728577

[ref32] Yamnik R. L. Digilova A. Davis D. C. Brodt Z. N. Murphy C. J. Holz M. K. (2009). S6 kinase 1 regulates estrogen receptor alpha in control of breast cancer cell proliferation. J. Biolumin. Chemilumin. 284, 6361–6369. 10.1074/jbc.M807532200, PMID: 19112174

[ref33] Yun C. Y. Choi H. You Y. J. Yang J. Y. Baek J. A. Cho E. S. (2016). Requirement of Smad4-mediated signaling in odontoblast differentiation and dentin matrix formation. Anat. Cell Biol. 49, 199–205. 10.5115/acb.2016.49.3.199, PMID: 27722013 PMC5052229

[ref34] Zhang X. Zhao J. Li C. Gao S. Qiu C. Liu P.. (2001). DSPP mutation in dentinogenesis imperfecta Shields type II. Nat. Genet. 27, 151–152. 10.1038/84765, PMID: 11175779

[ref35] Zoncu R. Efeyan A. Sabatini D. M. (2011). mTOR: from growth signal integration to cancer, diabetes and ageing. Nat. Rev. Mol. Cell Biol. 12, 21–35. 10.1038/nrm3025, PMID: 21157483 PMC3390257

